# The Aftermath of the COVID-19 Crisis in Saudi Arabia: Respiratory Rehabilitation Recommendations by Physical Therapists

**DOI:** 10.3390/healthcare9111560

**Published:** 2021-11-16

**Authors:** Ravi Shankar Reddy, Ajay Prashad Gautam, Jaya Shanker Tedla, Arthur Sá Ferreira, Luis Felipe Fonseca Reis, Kalyana Chakravarthy Bairapareddy, Venkata Nagaraj Kakaraparthi, Kumar Gular

**Affiliations:** 1 Department of Medical Rehabilitation Sciences, King Khalid University, Abha 61413, Saudi Arabia; rshankar@kku.edu.sa (R.S.R.); jtedla@kku.edu.sa (J.S.T.); vnraj@kku.edu.sa (V.N.K.); kmeny@kku.edu.sa (K.G.); 2 Postgraduate Program in Rehabilitation Sciences, Centro Universitário Augusto Motta, Rio de Janeiro 21032-060, Brazil; arthur_sf@icloud.com (A.S.F.); luisfelipefreis@gmail.com (L.F.F.R.); 3 Department of Physical Therapy, College of Health Sciences, University of Sharjah, Sharjah 27272, United Arab Emirates; kreddy@sharjah.ac.ae

**Keywords:** COVID-19, respiratory rehabilitation, physical therapy, critical care medicine

## Abstract

Since late 2019, the number of COVID-19 patients has gradually increased in certain regions as consecutive waves of infections hit countries. Whenever this wave hits the corresponding areas, the entire healthcare system must respond quickly to curb the diseases, morbidities, and mortalities in intensive care settings. The healthcare team involved in COVID-19 patients’ care must work tirelessly without having breaks. Our understanding of COVID-19 is limited as new challenges emerge with new COVID-19 variants appearing in different world regions. Though medical therapies are finding solutions to deal with the disease, there are few recommendations for respiratory rehabilitation therapies. A group of respiratory rehabilitation care professionals in Saudi Arabia and international experts have agreed with the World Health bodies such as the World Health Organization (WHO) on the treatment and rehabilitation of patients with COVID-19. Professionals participating in COVID-19 patient treatment, rehabilitation, and recovery formulated respiratory rehabilitation guidelines based on the DELPHI Method, combining scientific research and personal practical experience. As a result, it is envisaged that the number of individuals in the region suffering from respiratory ailments due to post-COVID-19 will decrease. This narrative review and clinical expertise guidelines may give physiotherapists acceptable and standard clinical guideline protocols for treating COVID-19 patients.

## 1. Introduction

The COVID-19 disease appeared in China as acute pneumonia in December 2019 [1]. COVID-19-affected patients presented with fever, breathing difficulty, cough, and a few patients presented with severe pneumonia symptoms [2]. People with immunodeficiency or chronic diseases (e.g., cancer, diabetes, lung diseases) or the elderly are prone to infections and more severe complications [3]. Individual care and well-being may also impact the likelihood of the new coronavirus attachment and its severity [2]. In this pandemic, those aged between 45 and 50 are more likely to be afflicted by the virus [4].

COVID-19 primarily attacks respiratory health, making it difficult for the patient to breathe [5]. Individuals infected with COVID-19 can develop an influenza-like respiratory tract infection, characterized by symptoms such as pyrexia (89%), tussis (68%), exhaustion (38%), phlegm production (34%), and difficulty breathing (34%) [6]. The severity of the disease varies from no symptoms to the worst form of pneumonia, resulting in respiratory failure or death [7]. According to recent reports, the majority of cases are either asymptomatic or mildly affected (≈80%); ≈15% are severely affected (require oxygen); ≈5% are critically affected (requiring ventilator and life support) [7].

Respiratory rehabilitation is a comprehensive therapy approach focused on individualized assessment and treatment to improve or maintain people with pulmonary diseases’ physical, social, and mental health [8,9]. It includes fitness training, education, and lifestyle modification, and aims to improve pulmonary disease patients’ overall health [8,10]. Physical therapy techniques are particularly relevant in the rehabilitation of patients with COVID-19 [9,11].

International organizations have attempted to recommend respiratory rehabilitation in their own countries [9,10,11]. Therefore, there was a need to establish standard respiratory rehabilitation guidelines for COVID-19 care in Saudi Arabia. Based on our clinical experience after dealing with several COVID-19 patients and through a literature search on respiratory rehabilitation, we propose these guidelines for physiotherapists involved in treating and managing COVID-19 patients in Saudi Arabia. These are the opinions of respiratory rehabilitation experts and experts from other disciplines summoned to deal with the crisis.

## 2. Methodology

**Figure 1:**
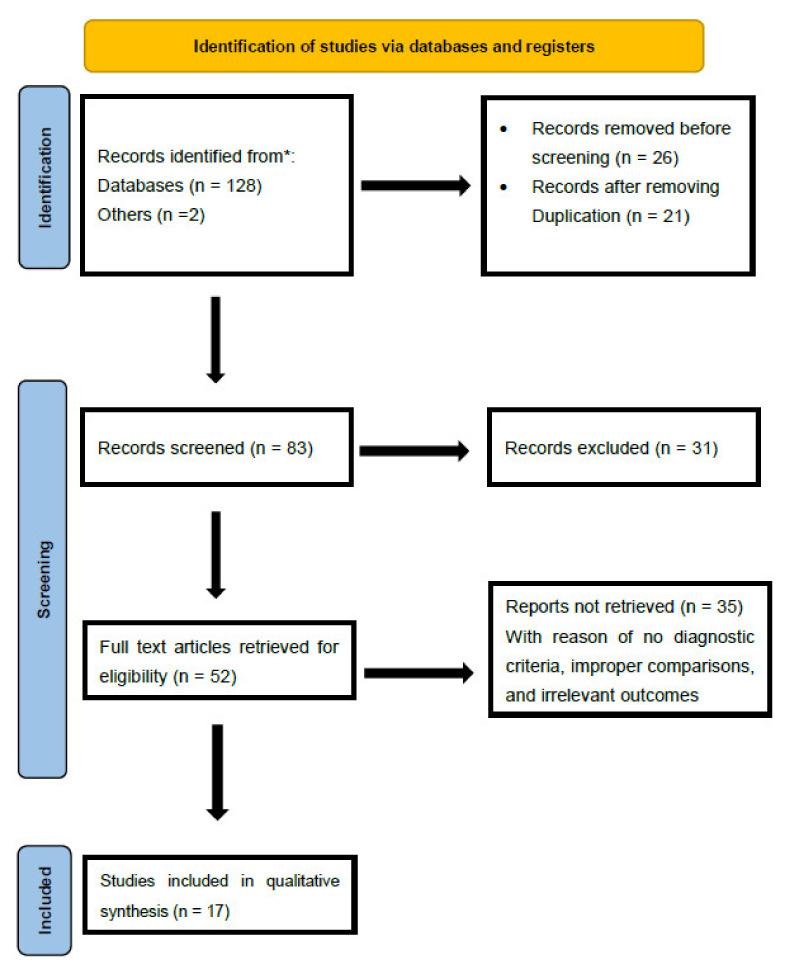
Flow diagram showing the review process and study synthesis. * Flow diagram showing the review process and study synthesis.

This narrative review focuses on respiratory rehabilitation guidelines for treating COVID-19 patients based on experts’ personal experiences and available scientific literature. The flow diagram shows the review process and the study synthesis by the experts (Figure 1). The studies included for clinical recommendations for COVID-19 physical therapy care by experts are presented in Table 1. A group of international cardiorespiratory physical therapy experts (*n* = 12) compiled the clinical recommendations. The expert group included seven clinical cardiopulmonary physical therapists and five academic physical therapy professionals. The first author organized a virtual forum to discuss the necessity for consensus for physical therapists to produce respiratory rehabilitation recommendations for COVID-19 patients. The author group first met virtually on April 30 2020, to discuss the immediate need for global acute care to manage COVID-19 patients and the need to develop respiratory rehabilitation practice guidelines. As a result, the task of developing scientific instructions for physical therapists in critical care settings was promptly prioritized.

### Literature Search

**Figure 2:**
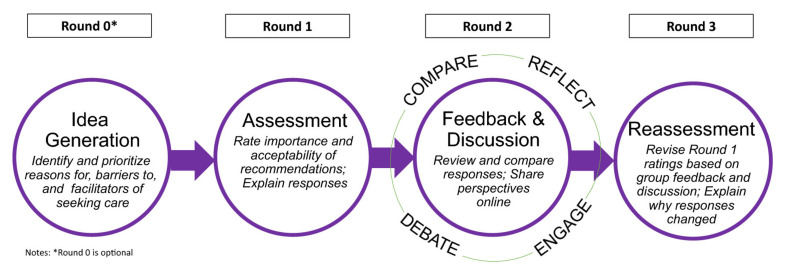
Delphi Method followed for common consensus and clinical recommendations by the expert panel. (Source: Khodyakov, Dmitry et al. “The RAND/PPMD Patient-Centeredness Method: a novel online approach to engaging patients and their representatives in guideline development.” 2019).

From creating databases from 30 April 2020, through 30 June 2021, we searched the published literature in Scopus, PubMed (Medline), Embase, Ovid databases, and other related scientific websites regarding respiratory rehabilitation and COVID-19 for any randomized clinical trials (RCT), systematic reviews, meta-analyses, or international recommendations and guidelines. The inclusion criteria included only articles that were written in English, and the articles’ full text could be retrieved and read. The exclusion criteria included articles that do not fall under the scope of rehabilitation or physical therapy and articles that are not related to patients (such as animal experiments or viral studies). The search terms included are: “COVID-19”, “new coronavirus pneumonia”, “acute respiratory distress syndrome (ARDS)”, “severe acute respiratory syndrome (SARS)”, “acute hypoxemic respiratory failure”, “influenza”, “Middle East respiratory syndrome (MERS)”, “pulmonary/respiratory rehabilitation or physical therapy and infectious diseases”, “non-invasive ventilation and acute hypoxemic respiratory failure”, “acute respiratory failure (ARDS)”, “mechanical ventilation”, and “weaning from mechanical ventilation.” The flow diagram shows the review process and the study synthesis by the experts (Figure 1). The recommendations are based on the published literature, and additional literature was consulted until all panel members agreed on the final draft of the respiratory rehabilitation guidelines. The panel virtually met on several occasions to formulate the recommendations, and the DELPHI method was followed to reach a common consensus (Figure 2).

**Table 1:** Studies for clinical recommendations for COVID-19 physical therapy care.

Reference	Title of the Study	Type of Research
Fila et al. (2021)	Recommendations for the respiratory rehabilitation of hospitalized and discharged COVID-19 patients: A systematic review	Review
Malyavin yet al. (2021)	Respiratory Rehabilitation for Post-COVID-19 Patients	Review
Zampogna et al. (2021)	Respiratory rehabilitation in patients recovering from COVID-19	Review
Vitacca et al. (2020)	Joint statement on the role of respiratory rehabilitation in the COVID-19 crisis: the Italian position paper	Protocol
Liu et al. (2020)	Respiratory rehabilitation in elderly patients with COVID-19: A randomized controlled study	Original Research
Poletti et al. (2020)	Respiratory Rehabilitation in the COVID-19 Era.	Review
Zhao al (2020)	Recommendations for respiratory rehabilitation in adults with coronavirus disease 2019	Review
Yan et al. (2020)	Effect of respiratory rehabilitation training on elderly patients with COVID-19: a protocol for systematic review and meta-analysis	Review
Zhu et al. (2020)	Effects of respiratory rehabilitation on patients with novel coronavirus (COVID-19) pneumonia in the rehabilitation phase: protocol for a systematic review and meta-analysis	Review
Thomas et al. (2020)	Physiotherapy management for COVID-19 in the acute hospital setting: clinical practice recommendations	Protocol
Righetti et al. (2020)	Physiotherapy Care of Patients with Coronavirus Disease 2019 (COVID-19)—A Brazilian Experience	Review
Cieloszczyk et al. (2021)	Recommendations for physiotherapyof adult patients with COVID-19	Review
Abdullahi et al. (2020)	Safety and efficacy of chest physiotherapy in patients with COVID-19: a critical review	Review
Lee et al. (2020)	Clinical course and physiotherapy intervention in 9 patients with COVID-19	Review
Polastri et al. (2020)	Recommendations from scientific/professional societies: an essential support for physiotherapy in patients with COVID-19	Protocol
Jiandani et al. (2020)	Evidence-based National Consensus: Recommendations for Physiotherapy Management in COVID-19 in Acute Care Indian Setup	Protocol
Abdullahi et al. (2020)	Physiotherapy management of COVID-19 in Africa: ongoing efforts, challenges, and future directions	Review

## 3. Precautions and General Recommendations for COVID-19 Patients

The World Health Organization (WHO) categorizes COVID-19 severity as (a) mild: patient with or without symptoms of severe pneumonia; (b) moderate: pyrexia, dyspnea, or suspected respiratory infection, a respiratory rate > 30 breaths/min, and SpO2 less than 90% in room air; and (c) severe: both side interstitial involvement on chest X-ray, PaO2/FiO2 < 300 [12,13,14].

In the absence of dedicated staff, healthcare professionals must clear the training test based on clear principles of behavior and action. In addition, respiratory rehabilitation providers must be appropriately skilled and experienced with respiratory physical therapy. All preventive measures must be implemented, and both operators and patients must wear all protective equipment stated in the relevant papers. RR is a three-phased systematic nonpharmacological therapy that includes assessment, treatment, and re-assessment (the evaluation is primarily functional, focusing on consciousness, pulmonary, cardiovascular, physical functions, and quality of life) [15]. The risk of droplet formation in the airways of a patient is very high. According to the WHO, to avoid or limit the possibility of this, all interventions and activities must be carried out—particularly concerning airway clearance interventions—with utmost care [16].

Whatever intervention mode, intensity, and timing are used, it must be tailored to each patient’s specific needs, especially for individuals with severe or critical conditions, the elderly, morbidly obese patients, comorbidities, and other complications [16]. Throughout the recovery phase, assessment and monitoring of COVID-19 patients should be continued [17]. Respiratory rehabilitation providers can also help patients cope with delirium, rage, fear, sleep disturbances, panic attacks, or a sensation of loneliness during isolation, and rigorous therapy and those at risk of non-compliance with treatment [18].

## 4. COVID-19 Respiratory Signs and Symptoms Are Discussed, as well as Possible Physical Therapy Referrals and Interventions

Mild symptoms without severe respiratory impairment, such as a fever and a dry cough, but no changes in a chest X-ray [19]. Treatment considerations: There will be no physical therapy contact with the patient, but telerehabilitation can be given for respiratory care advice [19]. Low oxygen demand (e.g., O_2_ flow of 5 liters/minute for maintaining SpO_2_ > 90%), cough with or without sputum production, and the ability to remove secretions autonomously are all characteristics of pneumonia [20]. Treatment considerations: There will be no physical therapy direct contact with the patient, but telerehabilitation can be given for respiratory care advice.

Mild symptoms with or without pneumonia characteristics such as comorbidity in the respiratory or neuromuscular systems (e.g., chronic obstructive pulmonary disease, myopathies or neuropathies, cystic fibrosis, bronchiectasis, spinal cord injury) [20]. Treatment considerations: Referral to physical therapy for airway clearing, and physical therapists must use airborne precautions. For non-ventilated patients, wearing a surgical mask during respiratory therapy sessions should be mandatory.

Mild disease with or without pneumonia symptoms, with signs of exudative secretions and ineffective self-secretion clearance capacity [20]. Treatment considerations: Referral to physical therapy with the intent of clearing the airway, and physical therapists must use airborne precautions. For non-ventilated patients, wearing a surgical mask during respiratory therapy sessions should be mandatory.

Severe disease suggests infections of the lower respiratory tract (e.g., increased O_2_ requirements; mild to high-grade fever; difficulty breathing or shortness of breath; severe, frequent, or productive or non-productive coughing episodes; radiological changes consistent with secretion accumulation) [20]. Treatment considerations: Referral to physical therapy for airway clearing, especially if the cough is ineffective and productive. There is radiological evidence of secretion accumulation. Physical therapists must take airborne precautions, and for the non-ventilated patient wearing a surgical mask during respiratory therapy sessions should be mandatory. It is recommended to optimize care as soon as possible and involve respiratory rehabilitation in the intensive care unit (ICU).

Mobilization, exercises, and rehabilitation [21]: If the patient is or with critical functional constraints, there is a high risk of developing problems such as frail or multiple comorbidity impairment of independence, for instance, in patients with ICU who experience a considerable decrease in functionality or at risk of ICU diseases, for example, mobilization, exercise, and rehabilitation. Treatment considerations for ICU-acquired patients: Referring to physical therapy, droplet precautions and precautions should be taken against airborne diseases when close contact is needed, or aerosol generation procedures may be necessary. For the non-ventilated patient, the patient must wear an operational mask throughout respiratory therapy sessions.

## 5. Physical Therapy for COVID-19 Individuals Who Are in the Critical Phase of the Disease

In conscious or unconscious patients, frequent posture changes, passive or active mobilization, and electrical stimulation (neuromuscular) are used to prevent disability [22]. Positional therapy with proper supervision is advised to maximize the V/Q ratio and avoid injury from immobility [22,23]. The prone position is suggested for several hours, but it must be discontinued if oxygenation deteriorates or if any significant problem arises. The trend in peripheral muscular strength changes should be assessed as soon as possible using appropriate tools.

The RR techniques in the critical phase must begin after the patient has attained some clinical stability to deal with ventilation and weaning. Treatments should be discontinued in the event of a high fever, increasing dyspnea, tachypnea >30 breaths/min, SpO_2_ <90% on O_2_ therapy, systemic arterial hypertension, respiratory distress, bradycardia, or tachycardia.

In individuals with minor bronchial obstruction, airway clearing procedures are not suggested during the acute phase [24]. Indeed, the possible benefits for operators do not justify the pollution concerns [24]. In patients with bronchiectasis or obvious bronchial burden, the risk/benefit ratio should be assessed on an individual case-by-case basis, employing techniques that ensure a safe distance from the patients [24].

Standard respiratory physical therapy protocols for dyspnea reduction, skeletal muscle training, tracheobronchial clearance, and activities of daily living training are not recommended since they can overburden the patient’s respiratory system and create distress [24]. It is recommended that ventilatory clinical parameters (cough, dyspnea, temperature, respiratory rate, SpO_2_, SpO_2_/FiO_2_, and thoracoabdominal dynamics) be evaluated twice a day.

## 6. COVID-19 Patients Receiving Physical Therapy Rehabilitation in the Acute Phase

Mobilization such as getting out of bed, making frequent postural changes, including continuous rotational therapy, asking patients to perform active upper and lower limb exercises, implementing muscle conditioning exercises, and strengthening upper and lower limb muscles are all recommended to prevent disability during this acute phase [24,25]. If patients have inspiratory muscular weakness, therapy includes activities to strengthen the muscles of the respiratory system [24]. Sedation may be used to relieve fatigue and dyspnea in patients with a non-productive dry cough [26]. Airway clearance procedures, ideally using single-use devices with self-management, are appropriate in excessive secreting patients with chronic pulmonary illnesses [26].

## 7. COVID-19 Patients Receiving Physical Therapy Rehabilitation in the Post-Acute Phase

For assessing peripheral muscle strength, manual or instrumental (e.g., handgrip dynamometer) muscle testing, isokinetic testing, and joint range assessments are recommended [27,28]. Reconditioning procedures are recommended for weaned patients and those on mechanical ventilation and oxygen for a long time to improve their physical functions and correct the motor and psychological effects of extended immobilization in the ICU [28]. Because the impact of viral infections on muscle activity is unknown, exercises are aimed at gradually increasing the load based on physical symptoms to maintain normal function [29]. Low-intensity exercises (≈3.0 MET) along with regular patient counseling and education are recommended [30]. A telehealth system can eventually perform rehabilitation programs for isolated individuals (i.e., educational videos, teleconsultation).

Patients who are discharged from hospitals should be given instructions on how to cope with physical activity and should be evaluated regularly in terms of function, capability, and involvement once they have been cured and no longer pose a risk of infection [30]. Balance evaluation is recommended for long-term bedridden patients [25]. A schedule should be established at the earliest possible time for evaluating the exercise capacity and O_2_ saturation response during efforts using walking tests and nighttime performance [25]. Patients who have recovered from severe COVID-19 frequently have physical and functional disabilities (such as changes in respiratory function, musculoskeletal and neurological function, limited participation in activities of daily living, and a decline in quality of life [31,32]. The severity of the normocapnic respiratory failure and the presence of physical and psychological dysfunction determine the length of time it takes to recover (anxiety, abandonment, depression, posttraumatic stress syndrome) [33]. It may take longer for patients with comorbidities to return to their previous status. Daily checks should be made for temperature, respiratory rate, cough, dyspnea, SpO_2_, SpO_2_/FiO_2_, and thoracoabdominal dynamics [33]. Regular and straightforward treatment regimens should be used for weaning oxygen therapy.

## 8. Specific Respiratory Physical Therapy Interventions

Bronchoscopy, invasive or non-invasive mechanical ventilation, intubations, and cardiopulmonary resuscitation are some of the procedures that produce aerosols when dealing with critical COVID-19 patients (in cardiac or respiratory failure) [34]. Treatment goals for oxygen therapy may differ based on the patient’s condition. Oxygen saturation, i.e., SpO_2_ > 92 to 96%, is the goal for severe hypoxia, respiratory distress, or shock patients [34,35]. When a patient is stable, the goal for SpO_2_ maintenance is > 92% in adults and approximately between 92–95% in pregnant females. The SpO_2_ aim should not be maintained over 96% in persons with COVID-19 and acute hypoxemic respiratory failure [35,36].

If treating therapists wear appropriate personal protective equipment (PPE), high-flow nasal oxygen (HFNO) and non-invasive ventilation is recommended for treating hypoxemic respiratory failure-related hypoxia because it has a limited risk of producing aerosols at flow rates of 40 to 60 L/min or with helmet and filters during non-invasive ventilation [37]. All these cases are required to be used in negative pressure rooms. When proper COVID-19 prevention measures are implemented, the risk of airborne transmission to employees is minimal [37,38]. Patients who require HFNO should use negative pressure chambers [39]. Only patients in airborne isolation rooms should be supplemented with HFNO. The flow rate should be kept at less than 30 liters/minute, which may help prevent viral transmission [39]. Despite the accumulated data in favor of the use of different non-invasive respiratory therapies for acute respiratory failure in COVID-19, it is not fully understood when to start, escalate, and de-escalate the best respiratory supportive option for the different timing of the disease. For many countries, non-invasive mechanical ventilation (NIMV) is not advised for routine use due to the significant hypoxic respiratory failure rate associated with COVID-19-related hypoxia [37,40,41]. However, evidence has gradually been consolidating in favor of non-invasive ventilatory strategies in the management of hypoxemic respiratory failure caused by COVID-19 [37].

Nebulized drugs such as salbutamol or similar types are not recommended for treating COVID-19 in non-intubated patients because they increase the risk of aerosol generation and infection spread to nearby health care professionals [42,43]. Wherever possible, metered-dose inhalers or spacers should be used. If a nebulizer is required, consult local standards to keep aerosolization to a minimum (e.g., using a Pari Sprint with an inline viral filter). Nebulizers, NIMV, HFNO, and spirometry should be avoided, and permission from senior medical professionals should be requested before using them. Airborne precautions should be used if they are deemed necessary. Although there is evidence favoring the prescription of some physical therapy techniques, some of these approaches are still recommended, such as postural drainage positioning, percussion, and vibrations, breathing techniques such as autogenic drainage, or active cycle breathing. Furthermore, several of these procedures, such as manual or mechanical hyperinflation, positive expiratory pressure (PEP) therapy, and mechanical insufflation-exsufflation, appear to be more effective and physiologically plausible for treating these patients [26]. Respiratory muscle training must be incorporated in various phases of rehabilitation to improve their strength and endurance [44]. Physiotherapists can also use inspiratory positive pressure breathing techniques in some instances, such as patients with rib fractures. NIMV can also be utilized for respiratory failure management or during exercise as part of airway clearance methods [45]. Airway suctioning and assisted or encouraged cough motions are two techniques that can help with secretion clearing [45,46,47].

## 9. Conclusions

Respiratory rehabilitation professionals in Saudi Arabia have been motivated to respond to the current COVID-19 pandemic. A group of physical therapy professionals involved in COVID-19 patient treatment, rehabilitation, and recovery developed respiratory rehabilitation guidelines based on the DELPHI Method, which combined scientific research with personal practical experience. To improve cardio-respiratory status, various physical therapy interventions have been found to be effective in different phases of COVID-19 patients’ care. For instance, in the acute phase/ICU, airway clearance and positioning are critical, whereas ambulation and respiratory muscle training are beneficial in the subacute phase. It is anticipated that these guidelines may help physiotherapists to effectively treat and manage COVID-19 patients with respiratory symptoms.

## Data Availability

On request to the corresponding author Ajay Prashad Gautam (agautam@kku.edu.sa), all data are available at the Department of Medical Rehabilitation Sciences.
